# Use of a Simple Ultrasound Device to Identify the Optimal Area of Compression for Out-of-Hospital Cardiac Arrest

**DOI:** 10.7759/cureus.12785

**Published:** 2021-01-19

**Authors:** Paul A Olszynski, Rhonda Bryce, Qasim Hussain, Stephanie Dunn, Brandon Blondeau, Paul Atkinson, Robert Woods

**Affiliations:** 1 Emergency Medicine, University of Saskatchewan, Saskatoon, CAN; 2 Clinical Research Support Unit, University of Saskatchewan, Saskatoon, CAN; 3 Faculty of Nursing, University of Regina, Saskatoon, CAN; 4 School of Health Sciences, Saskatchewan Polytechnic, Saskatoon, CAN; 5 Emergency Medicine, Saint John Regional Hospital, Saint John, CAN; 6 Emergency Medicine, Dalhousie, Halifax, CAN

**Keywords:** echocardiography, sonography, anatomy, resuscitation, cardiac arrest, chest compressions, cpr, ohca

## Abstract

Background

Despite automated defibrillation and compression-first resuscitation, out-of-hospital cardiac arrest (OHCA) survival remains low. Resuscitation guidelines recommend that chest compressions should be done over the lower half of the sternum, but evidence indicates that this is often associated with outflow obstruction. Emerging studies suggest that compression directly over the left ventricle (LV) may improve survival and outcomes, but rapid and reliable localization of the LV is a major obstacle for those first responding to OHCA. This study aimed to determine if a simplified, easy-to-use ultrasound device (bladder scanner) can reliably locate the heart when applied over the intercostal spaces of the anterior thorax in supine patients. Furthermore, we sought to describe the association between largest scan volumes and underlying cardiac anatomy with particular attention to the long axis of the LV.

Methodology

We recruited healthy male and female volunteers over 40 years of age. Using a bladder scanner to evaluate the left sternal border and mid-clavicular lines, we determined the maximal scan volumes at 10 intercostal spaces for each participant. Cardiac ultrasound was then used to evaluate the corresponding underlying cardiac anatomy and determine the area overlying the long-axis view of the LV. Descriptive statistics (means with standard deviations [SD], medians with interquartile ranges, and frequencies with proportions) were used to quantify demographic information, typical scan volumes across the chest, the frequencies of the best long-axis LV view location. This was then repeated for left sternal border assessments only. Kappa was determined when evaluating agreement between the largest left sternal border scan volume and the best long-axis LV view location.

Results

The long-axis LV was the predominant structure underlying the largest scan volume in 39/51 (76.5%) patients. When limited to left sternal border volumes only, the long axis of the LV was underlying the maximum volume intercostal space in 46/51 (90.2%; 95% confidence interval [CI]: 78.6%, 96.7%). The largest left sternal border scan volumes were located over the best long-axis LV view in 39/51 (76.5%, 95% CI: 62.5%, 87.2%) of the study participants with a Kappa statistic of 0.68 (95% CI: 0.52, 0.84; p < 0.0001).

Conclusions

In this cross-sectional study of healthy volunteers, an easy-to-use ultrasound device (bladder scanner) was able to reliably localize the heart. Largest scan volumes over the left sternal border showed substantial agreement with the intercostal space overlying the long axis of the LV. Further investigations are warranted to determine if such localization is reliable in cardiac arrest patients.

## Introduction

Despite automated defibrillation and compression-first resuscitation, out-of-hospital cardiac arrest (OHCA) survival remains low at approximately 10% [1-3]. Chest compressions during cardiopulmonary resuscitation (CPR) play a central role in the survival of patients in whom a shockable rhythm is not identified. According to the CPR guidelines of the American Heart Association [3], compressions should be performed midline over an area of compression defined by the lower half of the sternum. During CPR, forward blood flow results from a combination of effects on the thoracic cavity and heart, generating flow through what are postulated as the thoracic and cardiac pumps, respectively [4-6]. However, emerging research [7-11] suggests that compressions over the current area of compression (lower half of the sternum) often result in outflow obstruction that may significantly limit or even compromise pump effect. Compressions directly over the left ventricle (LV) may improve survival; however, rapid and accurate localization of the LV remains a major challenge, especially in OHCA.

A recent porcine study [12] reported promising improvements when compressions were performed directly over the LV as identified through the parasternal long-axis view, with a dramatic difference (69% versus 0%) in return of spontaneous circulation. The optimal area of compression was predetermined by identifying the mid-LV in its long axis using transthoracic echocardiography (TTE), with the effect on compressions subsequently confirmed using transesophageal echocardiography (TEE) during CPR. In human cardiac arrest studies, TEE-guided CPR can improve compression quality by guiding compressions according to the visualized LV changes, including greatest LV compression [13,14] or alleviation of outflow obstruction [15]. Cardiac arrest guidelines for TEE [16-18] outline the potential benefit of targeting LV compression to improve perfusion and clinical outcomes. There are, however, significant limitations to this evidence, especially when considering OHCA, given the limited expertise and time to perform this scan in the pre-hospital environment [13-17]. A rapid scan by a simple, easy-to-use ultrasound device may prove to be the right balance in terms of minimal complexity and maximal benefit.

Bladder scanners are ultrasound devices used to identify bladder location and volume [19]. Information is displayed following a series of automated scans through several planes taken in the supra-pubic abdominal area. Various models calculate a volume and generate a visual representation of the bladder location on a two-dimensional grid or B-mode image. Bladder scanners are non-invasive, rapidly identify volume, and their use requires minimal training [20,21]. To our knowledge, there is no data on the performance of bladder scanners in localizing the human heart. While the bladder and heart are both fluid-filled structures and amenable to ultrasound imaging, the bladder fills slowly, whereas the heart is a dynamic organ that changes shape beat to beat. This significant difference, however, is not the case with patients who are in cardiac arrest. It is possible that a device similar to a bladder scanner could be developed to allow rapid localization of the heart and LV in OHCA, providing rescuers an ultrasound-guided area of compression.

The primary objective of this study was to determine if an easy-to-use ultrasound device (bladder scanner) could reliably locate the heart when applied to the chest of healthy adult volunteers. We also explored the association between the largest scan volumes and underlying cardiac anatomy with particular attention to the LV.

## Materials and methods

**Figure 1:**
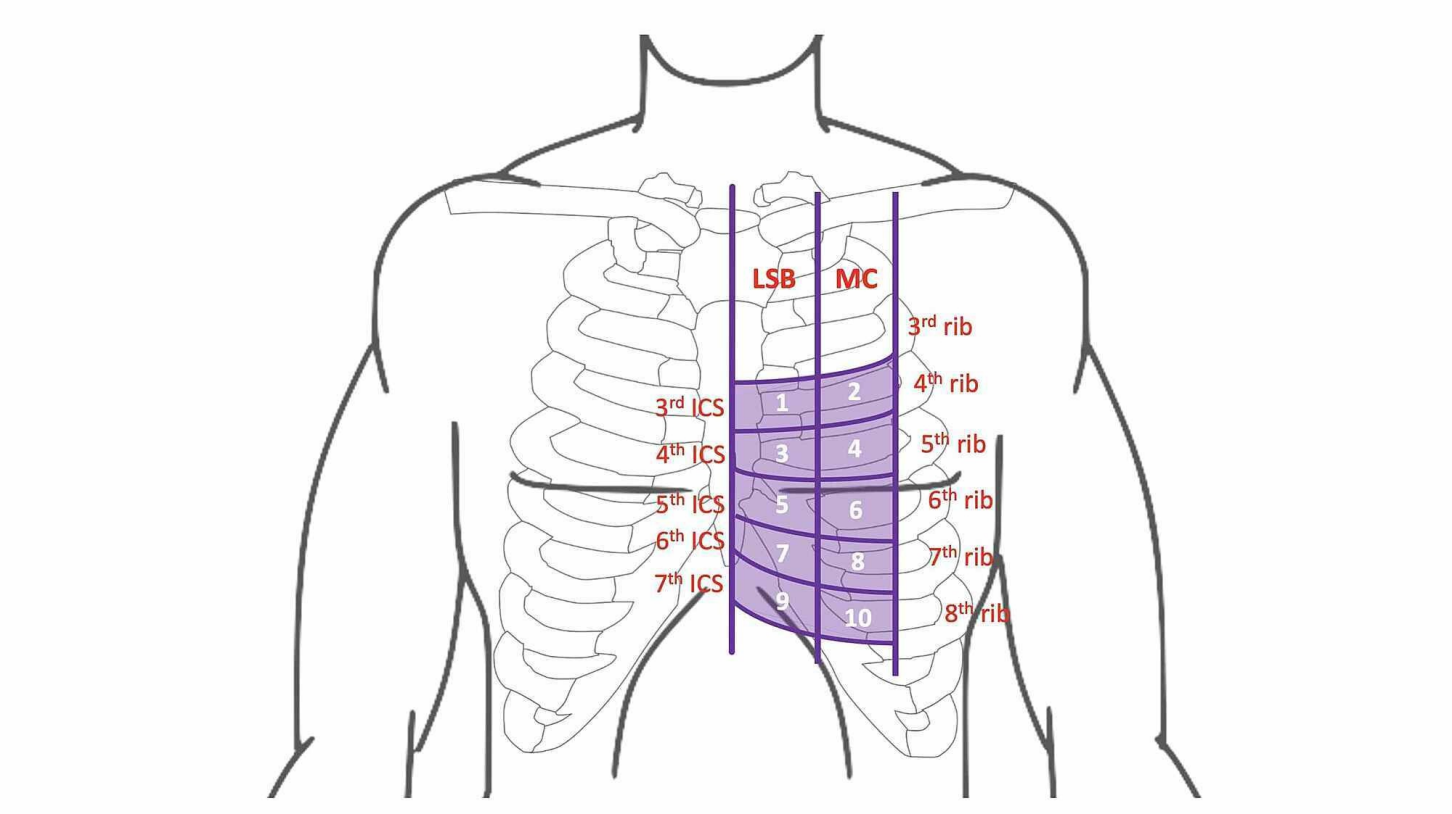
Ten-point interrogation grid (LSB zone, MC zone, and ICS) as defined by the patient’s ribs, sternum, and clavicle. LSB, left sternal border; MC, midclavicular; ICS, intercostal spaces

After obtaining approval from the Research Ethics Board of University of Saskatchewan (Bio #461), we recruited male and female volunteers over 40 years of age through the Clinical Learning and Resource Center’s Standardized Patient Program at the University of Saskatchewan. We collected biometric data including weight, height, and chest circumference at the nipple line. Body mass index (BMI) was calculated as weight (kg)/height (m)^2^ and classified as normal (<25), overweight (25-29.9), or obese (≥30). We pretested our scanning protocol (3 × 5 grid with the three longitudinal columns representing the left sternal border, mid-clavicular, and axillary zones, while intercostal spaces three to seven delineated the five rows) on a sample of 15 patients [22]. We then modified our scanning protocol by eliminating the axillary zone from our interrogation grid and by increasing the number of scans (volume) per space from two to four. We increased the number of scans (volume) at each intercostal space in an effort to capture a meaningful volume, speculating that variations were attributed to changes associated with cardiac cycle. In our final protocol, participants were placed in a supine position and a 2 × 5 grid was drawn onto their chests, with the two longitudinal columns representing the left sternal border and mid-clavicular zones, while five intercostal spaces (third to seventh) de-marked the rows for a total of 10 assessment spaces (Figure 1). For each space, the greatest of the four scan volumes was recorded as the value for that space.

**Figure 2:**
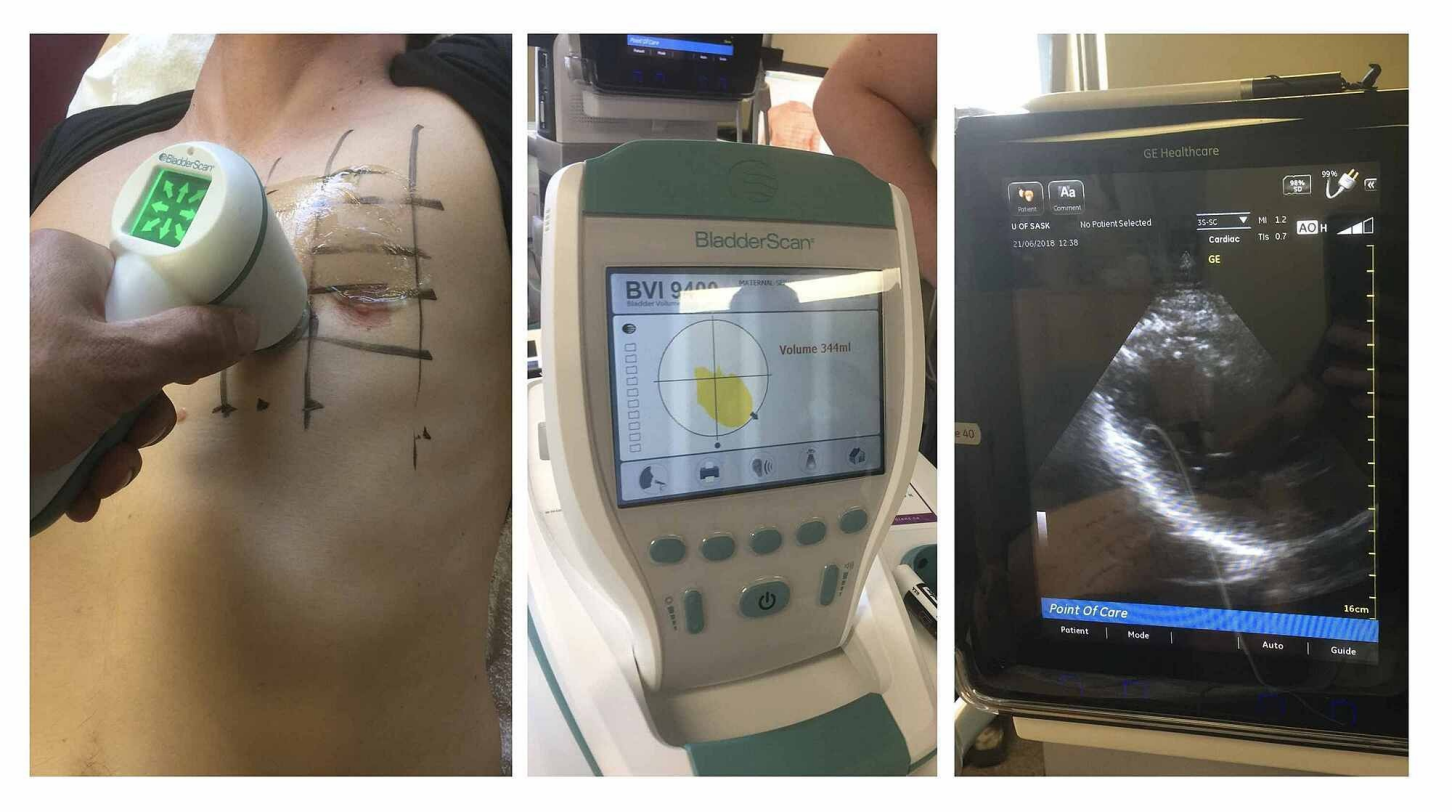
Scanning protocol.

Experimental localization of the heart was performed using a Verathon BladderScan Prime Plus bladder scanner (Verathon, WA, USA). After training during our pretest of 15 patients, each member of our inter-professional study team performed scans on several patients following a standardized approach and assessing each space on the grid. The transducer was placed perpendicular to the chest and volumes were recorded. This was followed by cardiac ultrasonography with GE Venue 40 machines (General Electric, NY, USA). Cardiac ultrasonography was done by PO and RW to determine whether the bladder scanner had correctly identified the heart, as well as to describe the underlying cardiac anatomy associated with the largest scan volumes (Figure 2). The ultrasound transducer was oriented along known echocardiographic windows, including the parasternal long axis, apical four-chamber, and subcostal views [23,24]. If a parasternal view of the heart was successfully obtained at the location of maximal detected volume, the space was deemed to be overlying either the long axis of the LV or aortic root (depending on which was the dominant structure in the view). If an apical four-chamber view was obtained, the space was deemed to be overlying the apex. If a subcostal view was obtained, the space was deemed to be overlying the inferior border of the heart (i.e., the right ventricle). When generating a parasternal view, if an adequate long-axis view of the LV was not achieved at the location of the maximum scanner volume, RW or PO performed cardiac ultrasound at the next largest scanner volume(s) until the best long-axis LV view was identified. Ultrasound was also used to describe other internal chest anatomy and included a scan for both pleural and pericardial effusions. All images were saved for subsequent review as necessary.

Descriptive statistics (means with standard deviations [SD], medians with interquartile ranges, and frequencies with proportions) were used to quantify demographic information, typical bladder scan volumes across the chest, and the frequencies of the best long-axis LV view location. This assessment also evaluated the proportion of scanner-determined intercostal spaces that were located over a significant portion of the long axis of the LV. Recognizing that assessment of 10 points during a resuscitation would be impractical, a repeat analysis was performed utilizing the largest scanner volumes only among the five left sternal border locations, again assessing the proportion of participants who would presumably receive LV compression if the maximal volume location was targeted. A second, more conservative evaluation was also undertaken to assess how frequently the predicted location by scanner volumes at the left sternal border overlay not just a substantial portion of the long axis of the LV but the best view of the LV. Both of these assessments limited to the left sternal border were further explored for differences by sex and BMI. To further quantify agreement between the ICS of the best LV view and the largest left sternal border volume, the associated Cohen’s Kappa statistic was calculated with its 95% confidence interval (CI). Proportions were compared between subgroups using Fisher’s exact test, given cells frequently had expected values less than five.

Interim analysis was planned at 50 patients (with original intent to recruit 100). Based on the analysis (results with associated CIs and Cohen’s Kappa statistic showing substantial agreement) and limitations of the current cohort (beating versus arrested hearts), the study team decided to conclude this study at 51 patients.

## Results

Demographic and anatomical details of participants are outlined in Table 1. The average participant age was 65.1 (SD = 9.9) years and 58.8% were females. Approximately three-quarters had a BMI at or exceeding 25 kg/m^2^. One participant had asthma and one participant had chronic obstructive pulmonary disease; no pleural or pericardial effusions were seen on imaging. The long-axis view of the LV was most commonly identified along intercostal spaces five to seven along the left sternal border (40/51, 78%).

**Table 1:** Demographic and anatomical characteristics of study participants (N = 51). y, year; SD, standard deviation; BMI, body mass index; LV, left ventricle; LSB, left sternal border; MC, mid-clavicular *Best long-axis LV view location missing for one patient

Age, y, mean (SD)		65.1	(9.9)
Sex, n (%)	Male	21	(41.2)
	Female	30	(58.8)
BMI, kg/m^2^, mean (SD)		28.9	(6.5)
BMI, kg/m^2^, n (%)	Normal (<25)	14	(27.5)
	Overweight (25–29.9)	20	(39.2)
	Obese (≥30)	17	(33.3)
Chest circumference, cm, mean (SD)	Overall	97.8	(11.6)
	Males	99.8	(10.0)
	Females	96.4	(12.7)
Long-axis LV view by intercostal space, n (%)*	3rd (LSB)	2	(4.0)
	4th (LSB)	7	(14.0)
	5th (LSB)	10	(20.0)
	5th (MC)	1	(2.0)
	6th (LSB)	20	(40.0)
	7th (LSB)	10	(20.0)

The largest scan volume reliably localized the heart in all participants (51/51); median scanner volumes determined at each of the 10 assessment points on the chest wall are presented in Table 2. When scanning both the left sternal border and mid-clavicular zones, the largest scanner volume correlated with the long axis of the LV in 39/51 (76.5%), the cardiac apex in 8/51 (15.7%), the aortic root in 3/51 (5.9%), and the right ventricle in 1/51 (2.0%). All seven individuals who had their largest volumes detected in the mid-clavicular zone were found to have their cardiac apex at this location.

**Table 2:** Scanner volumes (mls) detected by assessment location on 10-point chest wall grid, median (IQR). IQR, interquartile range; LSB, left sternal border; MCL, mid-clavicular line; ICS, intercostal space

ICS	LSB	MCL
3^rd^	0 (0, 82)	0 (0, 35)
4^th^	98 (34, 185)	0 (0, 55)
5^th^	144 (73, 187)	46 (0, 86)
6^th^	164 (110, 238)	41 (0, 128)
7^th^	107 (45, 186)	56 (0, 127)

When considering the largest volumes over the left sternal border exclusively, the long axis of the LV was the underlying cardiac structure in 46/51 (90.2%; 95% CI: 78.6%, 96.7%). Of the remaining five participants, the aortic root was the predominant underlying structure in three participants, and the cardiac apex and the right ventricle in the other two. In this revised approach, the seven individuals who previously had their largest scanner volume detected in the mid-clavicular zone, all of their largest left sternal border volumes were associated with views of the LV in its long axis (Table 3). The largest volume at the left sternal border was found to overlie the best long-axis view of the LV in 39/51 (76.5%, 95% CI: 62.5%, 87.2%) with a Kappa statistic of 0.68 (95% CI: 0.52, 0.84; p < 0.0001). Exploratory comparisons of the left sternal border volumes by sex and BMI did not identify any meaningful differences in either the proportions with the LV view beneath the maximal scanner volume or the proportion with the maximal scanner volume overlying the best LV view obtained.

**Table 3:** Frequency of scans with long-axis LV as the dominant underlying cardiac structure or best LV view beneath the largest LSB scan volume location. LV, left ventricle; LSB, left sternal border; ICS, intercostal space

Largest LSB scanner volume ICS			Long-axis LV in view at location		Best long-axis LV view observed at location		
ICS	n (%)		Yes, n	No, n	Yes, n	No, n	
3^rd^	3 (5.9)		3	0	2	1	
4^th^	6 (11.8)		6	0	4	2	
5^th^	9 (17.6)		8	1	7	2	
6^th^	22 (43.1)		19	3	17	5	
7^th^	11 (21.6)		10	1	9	2	
All	51 (100)		46	5	39	12	

## Discussion

Our findings suggest that the heart can be reliably localized using a simple, easy-to-use ultrasound device (bladder scanner). Our cardiac ultrasound findings reveal that the LV lies directly under the left sternal border across three intercostal spaces in the majority of our participants. Furthermore, the location of the largest scan volume along the left sternal border appears to correlate highly with the area overlying the long axis of the LV. As suggested by a previous porcine study [12], this represents a theoretically optimal area of compression as anteroposterior chest compressions at this location could result in LV compression across the long axis of the LV. We hypothesize that the correlation between the largest scan volume and the long-axis view of the LV may relate to the fact that the location of the parasternal long axis view provided the best acoustic window from which to determine volumes.

Radiological studies evaluating the location of critical cardiac structures have shown that midline compressions over the lower half of the sternum would compress the ascending aorta or the top of the left atrium 74% of the time [7], and that the LV is almost never the major underlying cardiac structure under the sternum at the inter-nipple line [9-11]. This is consistent with our findings. Proposals to move the recommended area of compression inferiorly and/or lateral to the left of the sternum have been made, but were met with reluctance. This is due to concerns about possible impairment of the thoracic pump effect, as well as concern for injury to intra-abdominal organs [9-11,25]; however, these concerns have not been demonstrated problematic in the limited animal studies or the emerging reports of TEE-guided compression [13-18].

We propose that compressions over the long axis of the LV, and specifically over the best long-axis view, may prove superior to compressions performed entirely away from the LV, which previous and emerging literature [7,9-11] suggests is likely the case with the current area of compression. Further studies on the mechanical effects of a compression along the left sternal border are warranted and may be facilitated using recently developed four-dimensional computed tomography [26].

Given the strong association between EMS-initiated or bystander CPR and increased survival, it is crucial to avoid delays in initiating chest compressions [1]. Much like the deployment of automated external defibrillators (AEDs) [27], it is conceivable that a simplified, ultrasound-based device could be developed to guide rescuers to the optimal area of compression. Such a device, when placed on the chest, would perform rapid sequential analysis of thoracic volumes along the left sternal border, resulting in a signal that informs rescuers where the largest volume has been detected and thus where compressions should be performed (this sonographic localization could take place immediately after rhythm analysis by the AED). The singular use of the left sternal border zone is predicated on the fact that in nearly all instances the left sternal border is where the long axis of the LV was identified (as per subsequent cardiac ultrasound). In instances where the largest volume was along the mid-clavicular zone, cardiac ultrasound revealed an apical four-chamber view. This implies that the scanner was closer to the apex, an area that is unlikely to result in effective compression of the LV.

Our study has several limitations. When the bladder scanner system is actuated, it analyzes the two-dimensional echoes from several sonographic planes and renders a three-dimensional reconstruction from which a volume is determined. Alnaif and Drutz [28] reported partial bladder volumes if the lateral borders were omitted, thus underestimating volumes. Given the dynamic nature of a beating heart, even with four distinct scans per space (with the greatest scan volume being recorded as the value for the space), our volumes have limited accuracy. That said, scanning a still heart (cardiac arrest) should be technically easier than a beating heart (as done in this study), likely negating the need for multiple scan attempts with the device. How this will impact accuracy remains to be determined in subsequent studies of patients who consent to be enrolled in such research upon their natural death. Furthermore, our sample size was relatively small, and participants were generally healthy with minimal intra-thoracic disease. Patients who suffer cardiac arrest have a higher prevalence of cardiac and thoracic pathology, including pleural and pericardial effusions, cardiomyopathies, valve and vascular abnormalities, and other entities that have the potential to significantly alter compression mechanics. Lastly, as we initiated our ultrasound search for the LV at the largest scan volume location and did not perform ultrasound scans in all 10 grid spaces, it is possible that we introduced bias in our interpretation of the LV location. Future studies should blind the two findings from each other until analysis.

## Conclusions

In this cross-sectional study, a simple ultrasound device (bladder scanner) was used to reliably localize the heart. In the healthy volunteers, the intercostal space of the largest scan volume was most often associated with the area overlying the long-axis view of the LV. This location, as identified through the scanner, represents a theoretically optimal area of compression. Further investigation into rapid sonographic localization of the heart to guide the area of compression for OHCA is warranted.
